# Correlation of increased corrected TIMI frame counts and the topographical extent of isolated coronary artery ectasia

**DOI:** 10.1186/s12872-018-0833-1

**Published:** 2018-05-22

**Authors:** Wei Wu, Shuyang Zhang, Yuchao Guo, Ruifeng Liu, Zhujun Shen, Xueqing Zhu, Zhenyu Liu

**Affiliations:** 1 Department of Cardiology, Peking Union Medical College & Chinese Academy of Medical Science, Peking Union Medical College Hospital, No.1 Shuai Fu Yuan, Beijing, 100730 China; 2 Department of Cardiology, The Second Affiliated Hospital of Zhejiang University School of Medicine, No. 88, Jiefang Road, Hangzhou, 310009 Zhejiang China; 3 Department of Cardiology, Beijing Friendship Hospital, Capital Medical University, No. 95 Yong An Road, Beijing, 100050 China

**Keywords:** Corrected TIMI frame count, Coronary artery ectasia, Slow flow, Topography

## Abstract

**Background:**

The precise relationship between increased thrombolysis in myocardial infarction (TIMI) frame counts and the topographical extent of isolated coronary artery ectasia (CAE) has not been fully explained. New parameters of linear dimension (LD) and the estimated ectatic area (EEA) together with the diameter and ectasia ratio may be associated with the corrected TIMI frame count (CTFC) in isolated CAE patients.

**Methods:**

The topographical parameters of ectatic coronary arteries and/or segments of 77 isolated CAE patients were consecutively studied. The CTFC for each coronary artery was determined by angiographic frame count.

**Results:**

Right coronary artery (RCA) was the most frequently affected. The RCA and left circumflex (LCX) had significantly longer LD (*p* < 0.001 for both), and greater EEA (*p* < 0.001 for both) than those of left anterior descending artery (LAD). Similarly, the RCA and LCX have higher CTFCs (*p* = 0.001 and *p* = 0.008, respectively) than LAD. All topographic parameters and CTFCs were positively correlated with Markis classification. Linear regression analyses revealed that CTFCs were strongly correlated with diameter, LD, ectasia ratio and EEA, while EEA was the best predictor for the CTFC. Among multiple linear and nonlinear regression models, the cubic model between the CTFC and EEA exhibits the best Goodness-of-Fit.

**Conclusion:**

The severity of the topographical extent of CAE was significantly correlated with increased CTFCs. Both the linear dimension and ectatic diameter (combined as EEA) were important for evaluating decreased coronary flow in isolated CAE patients.

**Electronic supplementary material:**

The online version of this article (10.1186/s12872-018-0833-1) contains supplementary material, which is available to authorized users.

## Background

Coronary artery ectasia (CAE) has been defined as the inappropriate dilatation of at least one coronary artery, with the luminal diameter being 1.5 times or more than that of adjacent normal segments [1, 2]. Although CAE was considered as being clinically less dangerous than obstructive coronary atherosclerosis, it could impair coronary flow [3, 4], induce myocardial ischaemia [5] and lead to severe vascular complications including myocardial infarction [2].

A high prevalence of slowed coronary flow in dilated coronary arteries has previously been reported. Thrombolysis in myocardial infarction (TIMI) frame count in CAE patients are significantly increased when compared with normal controls [3, 4, 6–9]. From a study of CAE patients in Turkey, patients with more than a 2.0-fold increase in maximum coronary diameter had higher TIMI frame counts than the 1.5- to 2.0-fold increase groups [8]. It was also indicated that patients with more dilated segments had slower coronary flow in the same cohort [9]. However, the precise correlation of increased TIMI frame count with the extension and severity of the ectasia has not been fully explained.

Our present study was designed to quantitatively analyse the correlations between the corrected TIMI frame count and the topographical parameters, including diameter, the ectasia ratio, the linear dimension of the ectatic coronary segment and the estimated area of ectasia. The study aims to provide more precise correlation of ectatic parameters to decreased coronary perfusion (increased coronary frame count) and tries to test the hypothesis that the more ectatic the coronary arteries are, the more severe the coronary flow will be affected.

## Methods

### Patients selection

A total of 6172 patients who underwent coronary angiography were consecutively and retrospectively recruited in the Department of Cardiology, at the Peking Union Medical College Hospital in China from January 2011 to October 2015. A total of 77 patients (58 males) were identified as having isolated coronary ectasia. Angiography was routinely performed by means of Judkins technique in multiple projections without intravenous nitroglycerin. Isolated CAE was defined as a localized or diffused coronary dilation that exceeded 1.5 times the diameter of the apparently normal segments without evidence of obvious stenosis (< 20%). In addition, patients with acute coronary syndrome were excluded since coronary flow assessment in these patients may be affected by their conditions.

### Angiography assessment

74 (96%) patients received transradial approach and the other 3 had transfemoral coronary angiographies. Contrast injection was done by manual operation in all patients. At least two experienced interventional cardiologists who were blinded to the patients’ basic status were required to make consentaneous diagnoses, quantitative coronary angiography (QCA) analyses and frame calculations on an off-line angiogram with the designated image processing software (SYNGO, Siemens, Germany). Mean values of the  parameters collected by different investigators were accepted.

### Parameter definitions

The corrected TIMI frame count (CTFC) was calculated as previously described [10]. Since the left anterior descending (LAD) artery TIMI frame count is approximately 1.7-times greater than that of the right coronary artery (RCA) and left circumflex artery (LCX), the TIMI frame counts of the LAD were adjusted by dividing by 1.7. If only one ectatic coronary artery was found, the artery was named the index vessel, and the CTFC collected from this vessel was considered the CTFC_index_. In cases with multivessel involvement, the maximal CTFC value of all ectatic vessels was considered the CTFC_index_, and the corresponding vessel was regarded as the index vessel. Additionally, the mean CTFC was defined as the mean value from the LAD, LCX, or RCA regardless of the existence of ectasia.

**Fig. 1:**
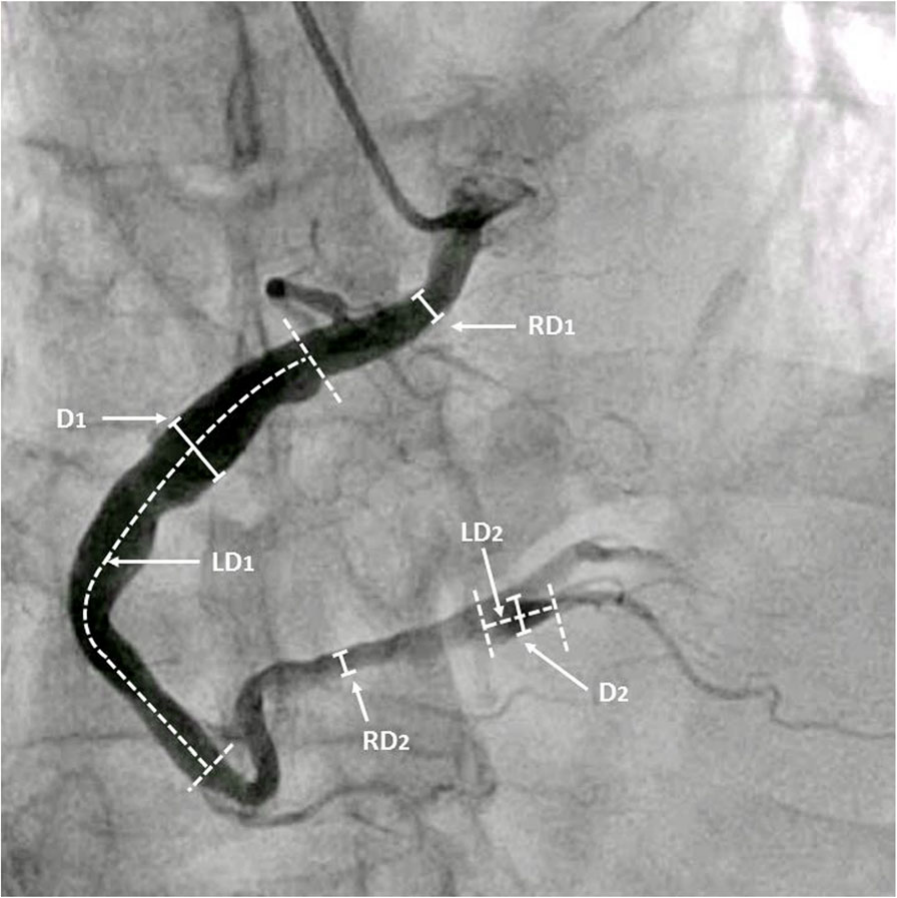
Definition of parameters measurement in an ectatic right coronary artery. Abbreviations: RD, reference diameter; D, maximal diameter; LD, linear dimension; LD_total_ = LD_1_ + LD_2_, EEA_1_ = D_1_× LD_1_, EEA_2_ = D_2_ × LD_2_, EEA_total_ = EEA_1_ + EEA_2_

The ectasia ratio (ER), maximal diameter (D), linear dimension (LD) and estimated ectatic area (EEA) acquired from QCA were applied as topographical parameters for further statistical analyses. The ER is defined as the maximal ectatic segment diameter divided by the normal adjacent segment diameter, in which the ER_index_ referred to the ER that was derived from the index vessel. Since the lumen diameters of proximal segment of normal LAD, LCX and RCA are 3.7 mm, 3.4 mm and 3.9 mm respectively [11] and Asian population have relatively smaller coronary artery size (about 0.22 mm) than Caucasian [12], a reference diameter of 3.5 mm was adopted when the ectasia lesions were so extensive that the adjacent reference segment was not applicable. It is also based on our centre’s data on Chinese population. The D_index_ and LD_index_ indicated the diameter and linear dimensions of involved segments of the index vessel as well as the LD_total_ indicated the full linear dimension of all involved segments of the three major vessels. To avoid complexity in calculation due to the irregularity of the shape of the dilated arteries, we simplified the calculation of the EEA by multiplying the diameter by the LD. In this sense, the EEA from the index vessel was calculated by the following equation: (EEA_index_) = D_index_ × LD_index_; and the total EEA from all vessels was calculated by the following equation: (EEA_total_) = EEA_1_ + EEA_2_ + … + EEA_n_ (n = number of involved ectatic segments). The parameter assessment of ectatic coronary artery was illustrated in Fig. 1.

### Statistical analysis

For statistical analyses, patients were divided into groups by the generally accepted Markis class [13], by different vessels, and by whether the patient presented with or without ectasia. All analyses were performed with the SPSS 19.0 statistical program (Statistical Package for the Social Sciences, SPSS Inc., Chicago, IL, USA). Continuous variables are expressed as the mean ± SD, and categorical variables are expressed as percentages. We determined whether each variable was normally distributed using the Kolmogorov-Smirnov test before statistical testing. Inter-group significant differences were tested by t-test or one-way analysis of variance (ANOVA) using Dunnett T3 methods for multiple comparisons (according to the heterogeneity of all variances), and McNemar test was used for categorical data according to certain circumstances. Applied statistical methods were illustrated in the legends of related tables or figures. The association between continuous and/or categorical topographical parameters were tested by Pearson correlation analysis and Spearman’s rank correlation analysis.

Linear regression models were deployed to study correlations between the CTFC_index_ and the topographic parameters of index vessels. CTFC_index_ was considered as the dependent variable, while D_index_, ER_index_, LD_index_ and EEA_index_ were regarded as independent variables. The EEA_index_ was regarded as the only independent variable in nonlinear regression models because the EEA contained more topographical information and exhibited a greater influence on the CTFC in the analysis. A two-tailed *P* value < 0.05 was considered statistically significant. The adjusted Ra square (Ra^2^) and Akaike information criterion (AIC) were used to quantify the Goodness-of-Fit in the different statistical models [14]. A larger Ra^2^ and smaller AIC suggested better statistical models.

## Results

### Baseline characteristics

From 6172 consecutive patients who received coronary angiography from our single-centre cardiac catheterization laboratory, a total of 77 patients (58 men, mean age 57.8 years old) with isolated coronary artery ectasia were retrospectively reviewed and included in this study. The clinical and angiographic characteristics of the study population are presented in Table 1. RCA has the highest prevalence of ectasia (66%) among the three major vessels. Mean CTFCs of the RCA and the LCX were significantly higher than that of the LAD (*P* = 0.001, *P* = 0.008, respectively).

**Table 1:** Clinical and Angiographic Characteristics of Isolated CAE Patients

	Isolated CAE *n* = 77	Inter-group comparison	*P*-value
Patient characteristics			
Age (years)	57.8 ± 9.6		
Male	58 (75%)		
Current smoker	21 (27%)		
Diabetes mellitus	22 (29%)		
Hypertension	46 (60%)		
Hypercholesterolemia	29 (38%)		
Markis class:			
I	16 (21%)		
II	23 (30%)		
III	21 (27%)		
IV	17 (22%)		
Vessel-specific characteristics			
Ectasia vessel:			
LAD	33 (43%)	LAD vs. LCX	0.109^a^
LCX	44 (57%)	LCX vs. RCA	0.401^a^
RCA	51 (66%)	RCA vs. LAD	0.007^a^
Mean value of maximal diameter(mm):			
LAD (*n* = 33)	4.53 ± 1.36	LAD vs. LCX	0.245^b^
LCX (*n* = 44)	4.83 ± 1.79	LCX vs. RCA	0.120^b^
RCA (*n* = 51)	5.30 ± 1.87	RCA vs. LAD	0.004^b^
Mean CTFC:			
LAD (n = 33)	28.2 ± 10.0	LAD vs. LCX	0.008^b^
LCX (n = 44)	32.9 ± 11.3	LCX vs. RCA	0.165^b^
RCA (n = 51)	36.1 ± 16.5	RCA vs. LAD	0.001^b^

*Abbreviations*: *CAE* Coronary artery ectasia, *LAD* Left anterior descending artery, *LCX* Left circumflex artery, *RCA* Right coronary artery, *CTFC* Corrected Thrombolysis in Myocardial Infarction frame count

^a^McNemar Test, ^b^T-Test

### Differences in CTFCs and topographical parameters by coronary distribution

**Fig. 2:**
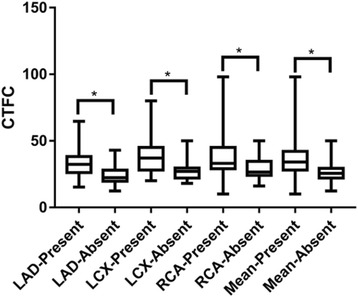
Comparison of CTFCs in coronary arteries with or without ectasia. Abbreviations: LAD, left anterior descending artery; LCX, left circumflex artery; RCA, right coronary artery; CTFC, corrected Thrombolysis in Myocardial Infarction frame count. * *P* < 0.001 by T test

**Fig. 3:**
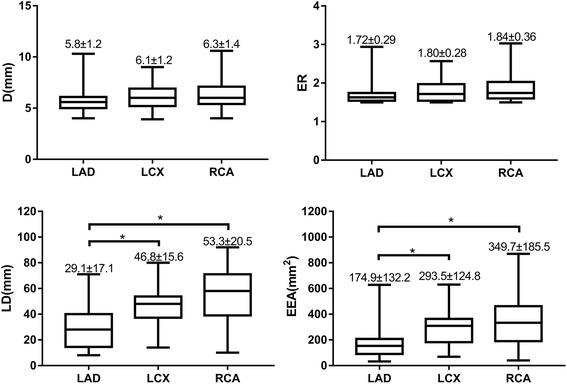
Topographical characteristics of different ectatic coronary arteries. Abbreviations: D, diameter; ER, ectatic ratio; LD, linear dimension; EEA estimated ectatic area. * *P* < 0.005 multiple comparisons from Dunnett T3 method

The CTFC of each artery with or without ectasia was compared In Fig. 2, and the topographical parameters in the ectatic LAD, LCX and RCA subgroups were compared in Fig. 3. In the isolated CAE population, the CTFCs of ectatic arteries (with CAE) were significantly higher than that of the normal ones (without CAE) in all three coronary arteries (LAD: 33.2 ± 10.8 vs. 24.4 ± 7.6; LCX: 37.3 ± 12.3 vs. 26.9 ± 6.0; RCA: 39.8 ± 18.4 vs. 28.8 ± 8.2; *P* < 0.001 for all, Fig. 2). For ectatic segments, the RCA and the LCX had a significantly longer linear dimension (53.3 ± 20.5 mm and 46.8 ± 15.6 mm vs. 29.1 ± 17.1 mm, *P* < 0.001, respectively) and a greater estimated ectatic area (349.7 ± 185.5 mm^2^ and 293.5 ± 124.8 mm^2^ vs. 174.9 ± 132.2 mm^2^, *P* < 0.001, respectively) than those of the LAD (Fig. 3).

### Differences in CTFCs and topographical parameters by the Markis classification

Mean CTFCs and the topographical parameters were compared by the Markis classification (Table 2). Since the Markis classification adopted the number of affected coronary arteries and the length of ectatic segments as benchmarks, it is reasonable that Markis type I patients have a greater ectatic linear dimension. As shown in Table 2, the maximal diameter, ectasia ratio and EEA were also significantly elevated as the severity increased from Markis classification IV to I (*P* < 0.001). The CTFCs exhibited the same pattern as the topographic parameters, and Markis type I patients had the highest CTFCs. Spearman’s rank correlation (Rs) analysis was used to assess the correlations between the Markis classification and the topographical parameters. It was observed that all of the studied topographic parameters had significant positive correlations with the Markis classification.

**Table 2:** CTFCs and Topographical Parameters Comparisons by Markis Types

	Markis IV (*n* = 17)	Markis III (*n* = 21)	Markis II (*n* = 23)	Markis I (*n* = 16)	*P*-value^a^	*Rs* ^*b*^
CTFC_index_	28.28 ± 4.83	36.8 ± 10.13	40.04 ± 11.62	56.35 ± 20.95	< 0.001	0.603
D_index_ (mm)	5.65 ± 0.85	6.16 ± 0.82	6.75 ± 1.01	7.15 ± 1.77	< 0.001	0.471
ER_index_	1.61 ± 0.16	1.78 ± 0.22	1.94 ± 0.28	2.07 ± 0.48	< 0.001	0.573
LD_index _(mm)	28.29 ± 7.33	58.57 ± 11.49	59.35 ± 14.17	63.63 ± 12.78	< 0.001	0.623
LD_total _(mm)	28.06 ± 7.34	58.57 ± 11.49	83.26 ± 14.45	131.94 ± 34.52	< 0.001	0.901
EEA_index _(mm^2^)	149.84 ± 36.68	361.81 ± 93.37	524.93 ± 126.62	893.99 ± 421.2	< 0.001	0.653
EEA_total _(mm^2^)	149.84 ± 36.68	361.81 ± 93.37	397.97 ± 123.92	456.36 ± 190.76	< 0.001	0.889

*Abbreviations*: *D* Diameter, *ER* Ectatic ratio, *LD* Linear dimension, *EEA* Estimated ectatic area

^a^One-way ANOVA, ^b^Spearman’s Rank

### Linear and nonlinear correlations of CTFCs and topographical parameters

**Fig. 4:**
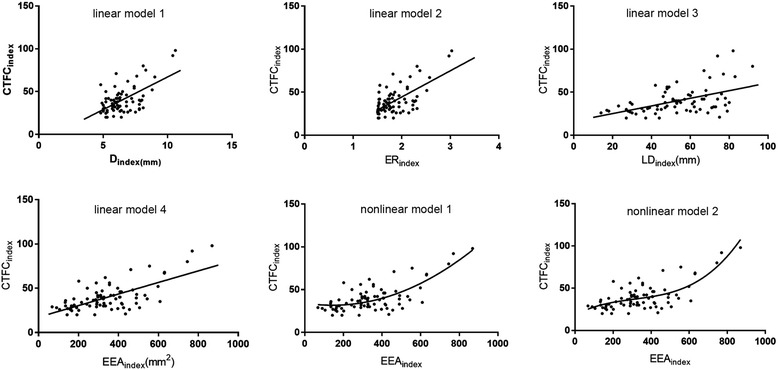
Linear and nonlinear regression correlations between CTFC_index_ and each of the topographical parameters. Correlation equations are showed in Table 3

The correlations between the CTFC_index_ values and the D_index_, ER_index_, LD_index_ and EEA_index_ were revealed through linear regression analyses (Table 3, Fig. 4). The CTFC_index_ was positively correlated with the D_index_, ER_index_, LD_index_ and EEA_index_ (Pearson correlation coefficient: 0.601, 0.646, 0.502 and 0.672, respectively; 2-tailed *P* < 0.001 for all parameters). The EEA_index_ had the largest Ra^2^, which suggested that the EEA_index_ was the best linear predictor for CTFCs in our study. Therefore, the EEA_index_ was selected as the sole parameter for further nonlinear regression model building, including quadratic, compound, growth, logarithmic, cubic, S, exponential, inverse, power and logistic models (SPSS 19, curve estimation) (Additional file 1: Table S1). We finally generated three multiple linear regression and two nonlinear regression models which had better Goodness-of-Fit than the previous linear regressions (Table 3). In the multiple linear regression equations, the model between CTFC_index_ and all four variables had a better Goodness-of-Fit (Table 3, Ra^2^ = 0.481; AIC = 378.7). In nonlinear regression models, the cubic model between the CTFC_index_ and EEA_index_ had a better Goodness-of-Fit (Ra^2^ = 0.546; AIC = 363.5).

**Table 3:** Linear, Multiple Linear and Non-linear Correlation Models of CTFC_index_ and Topographical Parameters

Model	Dependent Variable	Independent Variables	Equation	Ra^2^	AIC	RSD
Linear model						
1	CTFC _index_	D_index_	CTFC_index_ = −9.042 + 7.619* D_index_	0.353	392.9	12.6
2	CTFC _index_	ER_index_	CTFC_index_ = −16.246 + 30.404* ER_index_	0.410	385.7	12.0
3	CTFC _index_	LD_index_	CTFC_index_ = 16.360 + 0.444* L_index_	0.242	405.0	13.6
4	CTFC _index_	EEA_index_	CTFC = 17.407 + 0.065EEA_index_	0.444	381.1	11.6
Multiple linear model						
1	CTFC _index_	ER_index_ LD_index_	CTFC = −18.970 + 24.842ER_index_ + 0.245LD_index_	0.466	379.0	11.3
2	CTFC _index_	D_index_ LD_index_	CTFC = −14.396 + 0.285LD_index_ + 6.091D_index_	0.436	393.3	11.7
3	CTFC _index_	D_index_ ER_index_ LD_index_ EEA_index_	CTFC = 17.248-3.935D_index_ + 19.005ER_index_-0.354LD_index_ + 0.092EEA_index_	0.481	378.7	11.0
Nonlinear model						
1	CTFC _index_	EEA _index_	CTFC = 34.305-0.035EEA_index_ + 1.22^−4^EEA_index_^2	0.526	367.8	10.7
2	CTFC _index_	EEA _index_	CTFC = 18.208 + 0.122EEA_index_-2.94^−4^EEA_index_^2+ 3.13^−7^EEA^3	0.546	363.5	10.4

*Ra*^*2*^ Adjusted R square, *AIC* Akaike information criterion, *RSD* Residual standard deviation

## Discussions

It has been widely reported that isolated coronary artery ectasia was associated with increased TIMI frame counts, yet most of the previous studies have focused on the effect of the maximal diameter or the ectasia ratio of the involved coronary arteries on the TIMI frame count [8, 9]. It is interesting that in a group of patients with ectasia in the RCA alone, the TIMI frame counts of the LAD and LCX are higher than that in normal controls but lower than that of the RCA, which suggests that CAE is not a focal disease [15]. During our clinical practice, we noticed that in most cases, the more dilated the coronary artery was, the longer the ectatic segment turned out to be, and the linear dimension might have more effect on the decreased blood flow. To our knowledge, there has not been a previous study that analysed the relationship between the linear dimension of ectasia and the CTFC. To include more topographical information in one parameter, we also introduced the estimated ectatic area (EEA = D_index_ × LD) as a new parameter. This is the first study analysing the EEA together with other variables, including linear dimension, diameter, the ectasia ratio and the Markis type, to assess the extent of ectasia and to build a precise statistical model of the CTFC. One of the major findings of our study is that the topographical parameters of CAE, including the estimated ectatic area, ectasia ratio, diameter and linear dimension, are correlated with the corrected TIMI frame count. Additionally, CTFCs and topographical parameters differ in the three main coronary arteries.

According to the linear regression models, the EEA has the highest Ra^2^ and the smallest AIC among all topographical parameters, which supports its characteristic of being the best linear predictor of the CTFC in this group of CAE patients. Similarly, in our study, the CTFC was significantly higher when there were more diffuse ectasias and/or more vessels involved (Markis type IV to I), which is in accordance with previous reports [6, 16]. It suggests that the linear dimension may have a relatively similar effect as the ectasia ratio in disturbing normal coronary flow. The EEA, as a two-dimensional index, contains more information, including diameter and length, and could be a better parameter. However, since the real topographical extent of the ectatic segments involve irregular shapes in three dimensions, the estimated ectatic area could only partly simulate or represent the integrated profile. On the other hand, we also established precise statistical models of the topographical extent of the CAE and CTFC. The cubic model between EEA and CTFC has the best Goodness-of-Fit. The importance of the EEA in evaluating the ectatic extent of coronary arteries has been reinforced. In other words, the composite topographical factors of ectasia may be more important and precise than each of the single factor in predicting impaired coronary flow.

Decreased flow in ectatic arteries is likely the pathophysiological basis of ischaemic events in CAE patients, especially in isolated CAE patients [17]. The incidences of exercise-induced ischaemic symptoms, electrocardiogram changes or myocardium perfusion impairment are significantly higher in CAE patients than in normal controls [5, 18, 19]. In dilated coronary arteries, turbulent (back and forth) blood flow can be easily observed in the ectatic segment, and this phenomenon is usually attributed to endothelial dysfunction [20]. It is worth noticing that the increased CTFCs may indicate a high possibility of coronary thrombosis [17]. It is reported that the serum levels of plasminogen activator inhibitor-1, P-selectin, platelet factor-4 and D-dimer were increased [21, 22]. However, the fibrinolytic system was partly inhibited in patients with CAE, and disequilibrium of the coagulation/fibrinolytic system may be an important factor for micro or macro thrombotic coronary events [23]. All of the abovementioned factors lead to relatively poor myocardial perfusion and a high rate of myocardial ischaemic events.

Pathogenic and pathophysiological changes may simultaneously slow down coronary flow and alter the structure of coronary vascular walls. A high inflammatory status has been carefully studied in patients with CAE. It was reported that peripheral neutrophil counts and neutrophil-lymphocyte ratios were significantly higher in coronary ectasia patients [24, 25]. Inappropriate activation of neutrophils leads to increased levels of neutrophil elastase and neutrophil serine proteases, which increase the degradation of elastin fibres and the transition of collagen from type III to type I in coronary vascular walls [26, 27]. At the same time, significant endothelial dysfunction and high oxidative stress were also documented in patients with CAE [20, 28, 29]. All of these factors coexisted, and they all contributed to the slow coronary flow and destruction of coronary vessels. These may partly explain the close correlation between coronary ectasia and decreased coronary flow.

### Limitations

This study had several limitations. First, it was a single-centre observational study including a relatively small number of CAE patients; however, the occurrence of CAE is relatively rare, and approximately 30 patients were recruited in most of the previous reported studies. Second, we did not perform multivariate regression analysis to confirm the independent relationship between topographical extent of CAE and increased CTFC because of the limited sample size. Further investigations including more patients will be needed to confirm our findings. Third, the study population was limited to isolated CAE patients in order to exclude the influence of coexisting stenosis. However, CAE is usually complicated with coronary artery stenosis in clinical practice. Therefore, further studies regarding the effect of coexisting stenosis on CTFCs need to be carried out in a larger number of CAE patients.

## Conclusions

The present study suggests that the topographical extent of CAE is closely correlated with coronary blood flow. For the first time, we established a statistical association for the frame counts with parameters of dilatation and, especially, the estimated ectatic area. We suggest that the diameter and linear dimension of the ectatic coronary artery should be routinely reported in the angiographic findings of CAE patients.

## Additional file

Additional file 1: **Table S1.** Correlation Models of CTFC_index_ and Topological Parameters. (DOC 64 kb)

## Abbreviations

AIC: Akaike information criterion
CAE: Coronary artery ectasia
CTFC: Corrected TIMI frame count
D: Diameter
EEA: Estimated ectatic area
ER: Ectasia ratio
LAD: Left anterior descending artery
LCX: Left circumflex artery
LD: Linear dimension
RCA: Right coronary artery
TIMI: Thrombolysis in myocardial infarction
