# Supplementary material for: Correlation of increased corrected TIMI frame counts and the topographical extent of isolated coronary artery ectasia
Source: BMC Cardiovasc Disord. 2018 May 22;18:102. doi: 10.1186/s12872-018-0833-1 (PMC5964898; doi:10.1186/s12872-018-0833-1)
Supplement: Supplementary file 1 — Table S1. Correlation Models of CTFCindex and Topological Parameters. (DOC 64 kb) [file 12872_2018_833_MOESM1_ESM.doc]

**Table S1. Linear, Multilinear and Nonlinear Correlation Models of CTFC_index_ and Topographic** **Parameters.**

| Model | Dependent Variable | Independent Variables | Equation | Ra^2^ | AIC |
| --- | --- | --- | --- | --- | --- |
| Linear model | | | | | |
| 1 | CTFC_index_ | D_max_ | CTFC_index_=-9.042+7.619* D_max_ | 0.353 | 392.9 |
| 2 | CTFC_index_ | ER_index_ | CTFC_index_=-16.246+30.404* ER_index_ | 0.410 | 385.7 |
| 3 | CTFC_index_ | L_index_ | CTFC_index_=16.360+0.444* L_index_ | 0.242 | 405.0 |
| 4 | CTFC_index_ | EEA_index_ | CTFC=17.407+0.065EEA_index_ | 0.444 | 381.1 |
| 5 | CTFC_index_ | ER_index_ LD_index_ | CTFC=-18.970+24.842ER_index_+0.245LD_index_ | 0.466 | 379.0 |
| 6 | CTFC_index_ | D_index_ LD_index_ | CTFC=-14.396+0.285LD_index_+6.091D_index_ | 0.436 | 393.3 |
| 7 | CTFC_index_ | D_index_ ER_index_  LD_index_ EEA_index_ | CTFC=17.248-3.935D_index_+19.005ER_index_-0.354LD_index_+0.092EEA_index_ | 0.481 | 378.7 |
| Nonlinear model | | | | | |
| 1 | CTFC_index_ | EEA_index_ | CTFC=34.305-0.035EEA_index_+ 1.22^-4^EEA_index_^2 | 0.526 | 367.8 |
| 2 | CTFC_index_ | EEA_index_ | CTFC=18.208+0.122EEA_index_-2.94^-4^EEA_index_^2+ 3.13^-7^EEA^3 | 0.546 | 363.5 |
| 3 | CTFC_index_ | EEA_index_ | CTFC_index_=-60.817+17.591ln( EEA_index_) | 0.315 | 397.3 |
| 4 | CTFC_index_ | EEA_index_ | ln(CTFC_index_)=23.608+0.001EEA_index_ | 0.388 | -199 |
| 5 | CTFC_index_ | D_max_ | CTFC=100.237-24.380 D_max_ +2.251D_max_ ^2 | 0.455 | 379.9 |
| 6 | CTFC_index_ | D_max_ | CTFC=44.067-1.166D_max_^2+ 0.154 D_max_ ^3 | 0.450 | 379.3 |
| 7 | CTFC_index_ | D_max_ | CTFC_index_=-49.885+48.714ln( D_max_ ) | 0.307 | 398.2 |
| 8 | CTFC_index_ | D_max_ | ln(CTFC_index_)=14.120+0.152D_max_ | 0.289 | -188 |
| 9 | CTFC_index_ | ER_index_ | CTFC=87.632-73.617ER_index_ +25.602ER_index_ ^2 | 0.468 | 376.7 |
| 10 | CTFC_index_ | ER_index_ | CTFC=38.251-10.519ER_index_^2+5.561ER_index_ ^3 | 0.472 | 198.8 |
| 11 | CTFC_index_ | ER_index_ | CTFC_index_=-5.008+58.253ln( ER_index_ ) | 0.373 | 390.4 |
| 12 | CTFC_index_ | ER_index_ | ln(CTFC_index_)=12.190+0.608 ER_index_ | 0.338 | -193 |
| 13 | CTFC_index_ | L_index_ | CTFC=30.240-0.1627L_index_ +0.006L_index_ ^2 | 0.251 | 403.1 |
| 14 | CTFC_index_ | L_index_ | CTFC=0.942+2.101L_index_-0.042L_index_^2+3.09*10^-4^1L_index_ ^3 | 0.263 | 400.8 |
| 15 | CTFC_index_ | L_index_ | CTFC_index_=-32.259+18.488ln(L_index_ ) | 0.206 | 408.6 |
| 16 | CTFC_index_ | L_index_ | ln(CTFC_index_)=22.320+0.00976L_index_ | 0.244 | -183 |

Ra2: adjusted R square; AIC: value of Akaike information criterion, AIC=n*ln(residual sum of squares)+2*(p+1)-n*ln(n). n=77
